# The fibrosis-cell death axis in heart failure

**DOI:** 10.1007/s10741-016-9536-9

**Published:** 2016-02-16

**Authors:** A. Piek, R. A. de Boer, H. H. W. Silljé

**Affiliations:** Department of Cardiology, University Medical Center Groningen, University of Groningen, Hanzeplein 1, 9713GZ Groningen, The Netherlands

**Keywords:** Fibrosis, Heart failure, Cardiomyocyte, Cell death, Fibroblast, Myofibroblast, TGFβ, Hypertrophy

## Abstract

Cardiac stress can induce morphological, structural and functional changes of the heart, referred to as cardiac remodeling. Myocardial infarction or sustained overload as a result of pathological causes such as hypertension or valve insufficiency may result in progressive remodeling and finally lead to heart failure (HF). Whereas pathological and physiological (exercise, pregnancy) overload both stimulate cardiomyocyte growth (hypertrophy), only pathological remodeling is characterized by increased deposition of extracellular matrix proteins, termed fibrosis, and loss of cardiomyocytes by necrosis, apoptosis and/or phagocytosis. HF is strongly associated with age, and cardiomyocyte loss and fibrosis are typical signs of the aging heart. Fibrosis results in stiffening of the heart, conductivity problems and reduced oxygen diffusion, and is associated with diminished ventricular function and arrhythmias. As a consequence, the workload of cardiomyocytes in the fibrotic heart is further augmented, whereas the physiological environment is becoming less favorable. This causes additional cardiomyocyte death and replacement of lost cardiomyocytes by fibrotic material, generating a vicious cycle of further decline of cardiac function. Breaking this fibrosis-cell death axis could halt further pathological and age-related cardiac regression and potentially reverse remodeling. In this review, we will describe the interaction between cardiac fibrosis, cardiomyocyte hypertrophy and cell death, and discuss potential strategies for tackling progressive cardiac remodeling and HF.

## Heart failure and cardiac remodeling

Fibrosis is the excessive deposition of extracellular matrix (ECM), such as collagens and fibronectin, resulting in the excessive accumulation of fibrous connective tissue [1, 2]. Fibrosis is an essential process in the repair of damaged tissues and wounds, but its accumulation in organs and tissues can lead to scarring, organ dysfunction and, ultimately, failure. In many chronic diseases, sustained progressive fibrosis can be very detrimental, like in fibrotic kidney and liver disease, and this is also true for chronic heart failure [3]. Heart failure (HF) is a complex clinical syndrome in which reduced cardiac function results in insufficient perfusion of peripheral tissues [4, 5]. HF is an enormous health problem in the Western society, in which more than 8 % of the population aged 75 years and older are diagnosed with HF, and prevalence and incidence rates are not expected to decline in the upcoming decade [6]. Cardiac remodeling, which can be described as any structural and functional change of the heart, underlies HF development. Cardiac remodeling is a reaction of the heart to reduce ventricular wall stress in response to changes in after load (pressure load), preload (volume overload) or myocardial injury [7]. Amongst others, main risk factors for HF include coronary artery disease and hypertension [6]. A recent study of a community-based cohort revealed that more than 70 % of new HF patients had a history of hypertension and more than 25 % have had a preceding myocardial infarction (MI) [8]. Regardless of the initiating events, cardiac hypertrophy, cardiomyocyte cell death and fibrosis constitute key features of pathological cardiac remodeling.

**Fig. 1:**
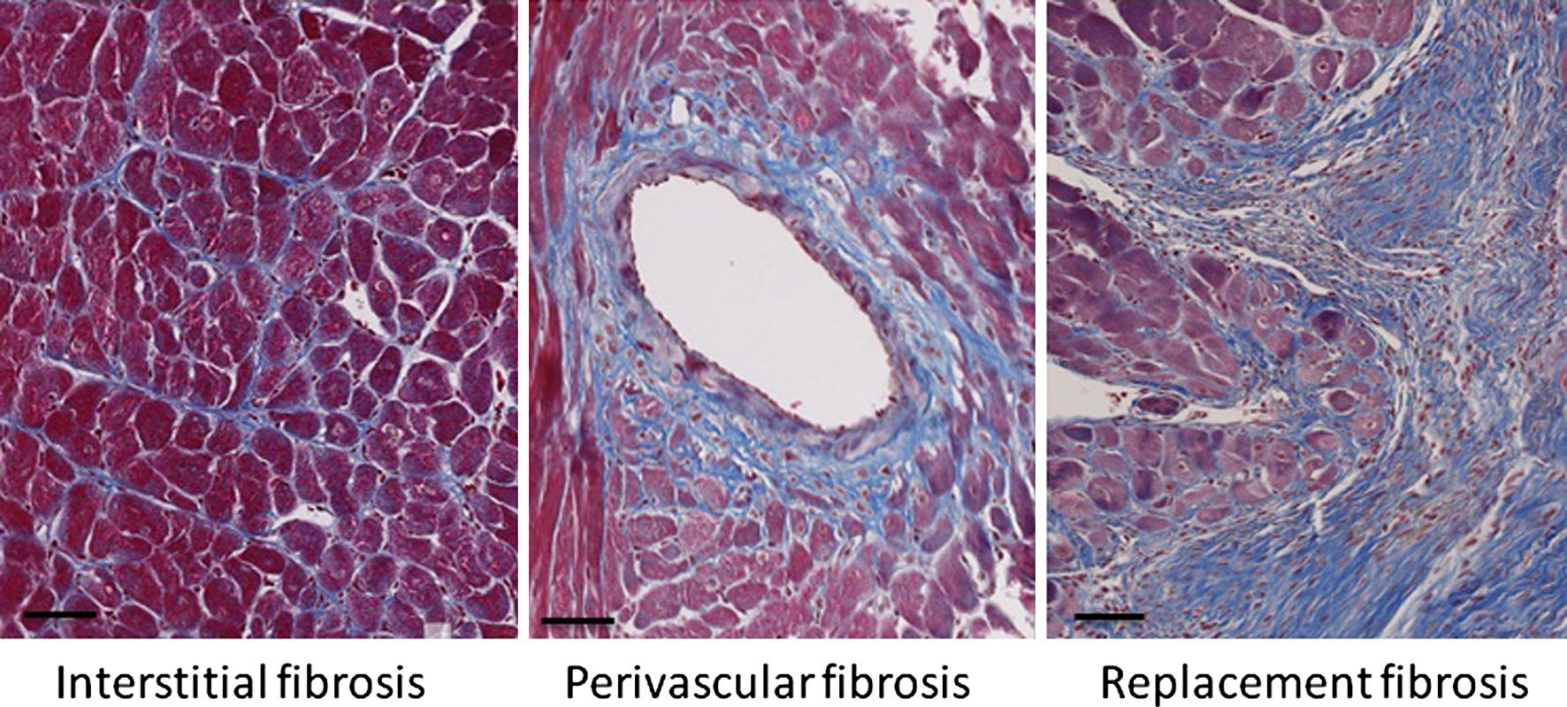
Different types of fibrosis. Mouse cardiac tissue was stained with Masson’s trichrome stain to visualize cardiomyocytes in *red* and fibrotic fibers in *blue*. Pictures with interstitial and perivascular fibrosis are from pressure overload (TAC) mouse hearts and replacement fibrosis from mouse hearts with a myocardial infarction. *Bar* indicates 50 µm

Fibrosis is a hallmark of pathological cardiac remodeling and is absent under physiological stress conditions, such as exercise and pregnancy [9–11]. Cardiac fibrosis appears to be an irreversible process [12] and is increasingly recognized as a major cause of morbidity and mortality in many chronic diseases. In the myocardium, fibrosis can be divided into interstitial fibrosis, replacement fibrosis and perivascular fibrosis, which all have their characteristics (Fig. 1). The distinction between different types of fibrosis is made based on cause and anatomical localization [13]. As a response to increased wall stress generated by a cardiac stressor, like hypertension, interstitial reactive fibrosis is developed in the myocardium [14, 15]. Reactive interstitial fibrosis is located in the ECM surrounding cells and is defined as the expansion of ECM without cardiomyocyte loss and is characterized by a widespread deposition of collagens throughout the myocardium [13, 14]. In perivascular fibrosis, which is also associated with hypertension, fibrillar collagens accumulate in the adventitia of intramural coronary arteries [14, 16, 17]. With ongoing hypertension, the accumulation of collagens progresses [17].

Replacement fibrosis occurs in the myocardium to replace dead tissue and is an essential repair process after myocardial infarction [13, 14, 16, 18]. Since cardiomyocytes have no or only very limited proliferative capacity, cardiomyocyte replacement does not occur in the damaged heart and scar formation is the only reparative mechanism to prevent cardiac rupture. Replacement fibrosis does also occur under other pathological conditions that will ultimately affect cardiomyocyte viability, like in sustained hypertension, cardiomyopathies and valve insufficiencies. This may result in a more dispersed fibrosis throughout the myocardium.

Thus, fibrosis may initially have cardiac preserving functions, but a sustained fibrotic response, as observed under pathological conditions, may negatively impact on cardiac function and finally exacerbate cardiac functional decline. Persistent accumulation of collagens disturbs tissue architecture, stiffens the heart and can affect both systolic and diastolic function and contribute to arrhythmias [17]. In agreement with this hypothesis, Hein et al. [19] observed in aortic stenosis patients with HF an increase in fibrosis and cell death. This was strongly increased in decompensated patients in whom fibrosis and myocyte degeneration was strongly accelerated [19]. These observations support the view that an apparent vicious cycle of impaired cardiac function, fibrogenesis and cell death exists and if not properly treated this cycle will accelerate and result in cardiac failure [19]. Not surprisingly, fibrosis-associated proteins, including soluble suppression of tumorigenicity 2 (sST2) and galectin-3 (Gal-3), which can be detected in blood plasma, are increasingly recognized as markers for poor prognosis of HF patients [20–23]. These biomarkers have recently been included in the AHA/ACC clinical guidelines for potential additive risk stratification of patients with established HF [5]. Nevertheless, additional research will be required to prove that circulating biomarkers properly reflect histological myocardial fibrosis [24].

**Fig. 2:**
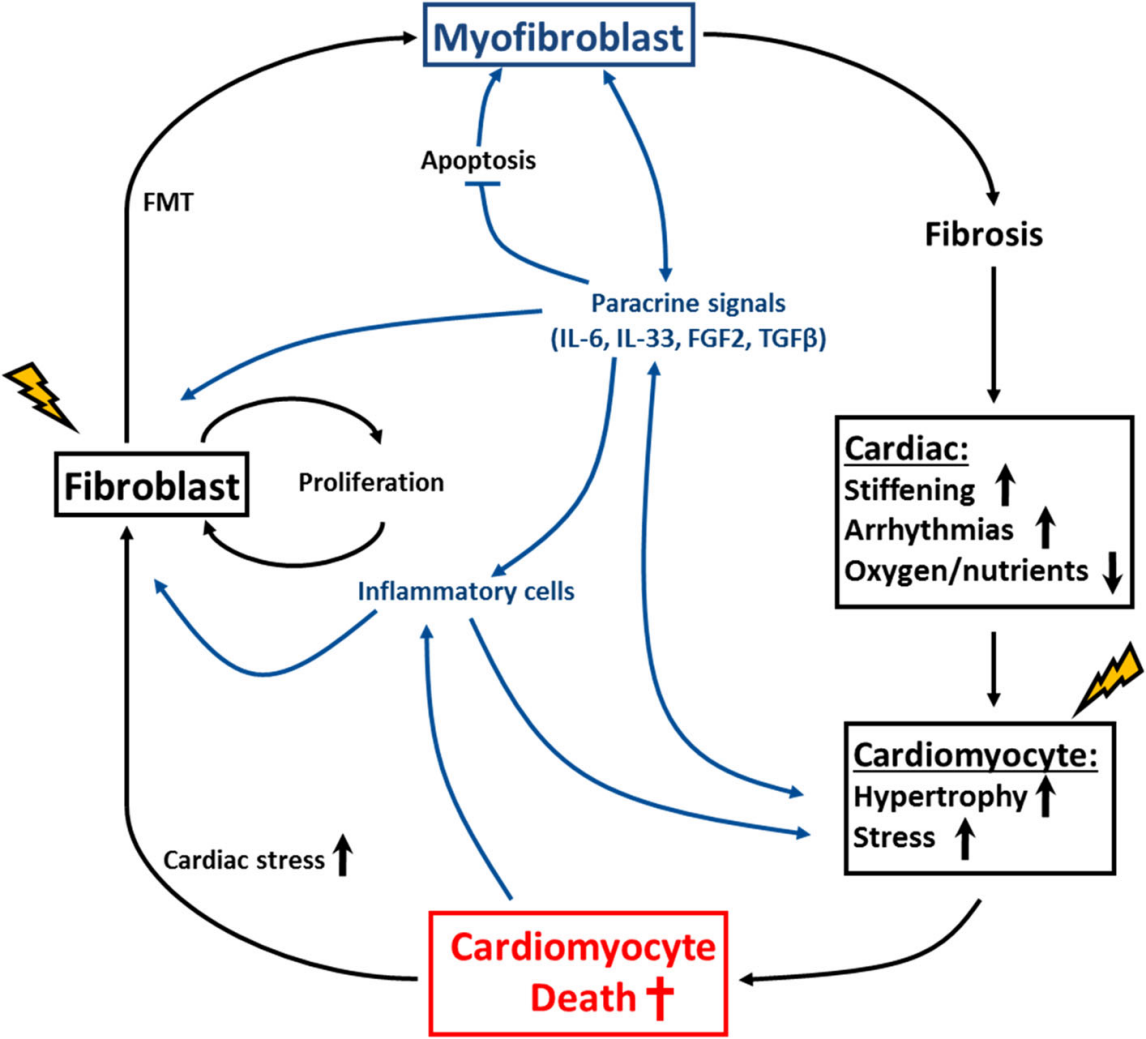
A simplified depiction of the vicious cycle of myofibroblast activation, impaired cardiac function and cardiomyocyte cell death. This simplified scheme shows in the *outer circle* (*black*) the role of myofibroblasts in fibrogenesis and cardiac impairment, whereas the inner processes predominantly reflect paracrine signaling effects. Together, this generates a sustained response of progressive cardiac deterioration. *FMT* fibroblasts to myofibroblasts transition, *IL*-*6* interleukin-6, *IL*-*33* interleukin-33, *FGF2* fibroblast growth factor 2, *TGFβ* transforming growth factor beta. *Lightning symbols* indicate stressors acting on the cells

In contrast to cardiac fibrosis, hypertrophy is generally believed to be an adaptive and protective mechanism since it also occurs in athletes and pregnant women [9–11]. This physiological hypertrophy is reversible and does not decompensate to HF. Sustained wall stress associated with cardiovascular diseases generates, however, a pathological form of hypertrophy, which is associated with reduced cardiomyocyte function. Among others, pathological hypertrophy involves alterations in Ca^2+^ handling, increased oxidative stress, changes in excitation–contraction coupling, sarcomere dysfunction and metabolic and energetic remodeling [9–11]. These cellular and molecular changes finally culminate into cardiomyocyte death caused by necrosis, apoptosis and/or phagocytosis [9, 25, 26]. Importantly, myofibroblasts, which are the main producers of ECM components, also secrete numerous factors with inflammatory and paracrine functions and play an essential role in hypertrophy development under pathological conditions. The contribution of myofibroblasts toward cardiac dysfunction is therefore twofold, namely via connective tissue formation and via production of paracrine and inflammatory molecules [27, 28]. Together, this will promote pathological cardiomyocyte hypertrophy and may finally lead to cardiomyocyte death and progressive deterioration of cardiac function. Excellent reviews have been published on apoptosis (programmed cell death), necrosis and phagocytosis, and we will not elaborate on these and other modes of cell death [26, 29–31]. Here, we will review the deleterious effects of activated (myo)fibroblast on cardiac function and postulate an apparent vicious cycle of fibrogenesis, impaired cardiac function and cardiomyocyte cell death (Fig. 2).

## Cardiac fibroblasts and myofibroblasts

In the healthy heart, normal amounts of fibrillar collagens form a network between different cell types within the myocardium and are essential to provide a scaffold for myofiber alignment and to maintain cardiac geometry during systole and diastole of the heart. Collagen I and III are the main collagens in the heart and constitute about 85 and 10 % of collagen, respectively [16]. Fibroblasts, which constitute the majority of the large (40–70 %) non-myocyte population in the heart [32–34] are responsible for the turnover of ECM proteins and play an essential role in maintaining the integrity of connective tissue. It is generally believed that most cardiac fibroblasts in the myocardium are derived from the pericardium during development, but proper fait mapping is still lacking [34]. Fibroblasts are a heterogeneous group of cells, and so far no specific markers have been identified for fibroblasts. Discoidin domain receptor 2 (DDR2), a tyrosine kinase cell surface receptor for collagen [35], has been predominantly used to define fibroblast in the heart [33]. DDR2 is also expressed in other cells outside the heart, and among others, it is important for chondrocyte proliferation [36]. Thus, although it may specify fibroblasts in the heart, it is not a specific fibroblast marker in general.

A common feature to fibrotic diseases is the activation of fibroblasts and their differentiation into myofibroblasts, which express and secrete much higher levels of ECM proteins. Myofibroblasts are not usually present in healthy cardiac tissue, with the exception of heart valve leaflets. Fibroblasts to myofibroblasts transition (FMT) can be induced by mechanical stress, cytokines, growth factors and ECM components [37]. Transforming growth factor β (TGFβ) is the most potent activator of FMT. Three homologues of TGFβ isoforms exist (TGFβ1, TGFβ2, TGFβ3) in mammalians that are produced as latent precursors bound to latency-associated peptide (LAP), which can interact with latent TGFβ-binding proteins (LTBP1–4) to form a large latent complex (LLC) that is effectively secreted into the extracellular space. Only after cleavage of this complex by extracellular proteases, TGFβ becomes liberated and can bind to its cellular receptor [38, 39]. TGFβ signals via a heteromeric tyrosine kinase complex of TGFβ type I receptor (TGFBR1, also termed Alk5) and type II receptor (TGFBR2), in which TGFBR2 phosphorylates TGFBR1, which propagates the signal via activation of SMAD transcription factors. This results in relocalization of the SMAD2/SMAD3 to the nucleus and the expression of ECM matrix proteins and alpha-smooth muscle actin (αSMA) [40]. The latter is used as a marker to identify myofibroblasts, although it is not specific and is also strongly expressed in smooth muscle cells [41]. αSMA allows contraction of myofibroblasts [42], and this is important during wound closure, but in the heart it can affect cardiac function and contribute to arrhythmogenicity [43]. Importantly, inhibition of TGFβ signaling has been shown to reduce fibrosis in animal models of cardiac remodeling and to preserve cardiac function [44, 45].

Besides FMT, TGFβ also stimulates epithelial mesenchymal transition (EMT) and endothelial mesenchymal transition (EndoMT) [46, 47]. It has been reported that approximately 27–35 % of all cardiac fibroblasts in the pressure-overloaded mouse heart (aortic banding) were derived from endothelial cells via EndoMT [48]. Inhibition of EndoMT by infusion of recombinant bone morphogenic protein-7 (BMP-7) reduced fibrosis in the mouse pressure-overloaded heart by almost 50 % [48]. Recent cell lineage tracking studies in mice reported much lower levels of EndoMT-derived fibroblasts and, moreover, did not provide a role for enhanced EndoMT during cardiac pressure overload [49, 50]. Thus, although EndoMT plays an important role during development of HF [51, 52], the role for EndoMT in the stressed heart is still controversial. Circulating bone marrow-derived cells have also been proposed to be a source of myofibroblasts in the diseased heart [53, 54], but cell lineage tracking has also disputed these results [49, 50].

Recently, Kramann et al. [55] identified glioma-associated oncogene family zinc finger 1 (Gli1) positive perivascular mesenchymal stem cells (MSCs) as a source of myofibroblast cells in mouse tissue after induction of organ damage, including the pressure-overloaded heart (aortic banding). Interestingly, ablation of these Gli1^+^ cells reduced cardiac fibrosis by almost 50 %, but also reduced cardiac hypertrophy and preserved left ventricular ejection fraction. This nicely exemplifies the importance of myofibroblasts in the fibrotic process and in the induction of pathological hypertrophy. The role of TGFβ in the transition of these Gli1^+^ cells is so far not known.

Thus, although the exact nature of myofibroblasts in the heart remains obscure, these cells are most likely generated from resident cell types in the heart. Inhibition of the formation of these myofibroblast populations reduces fibrosis and improves cardiac function.

## Fibrosis impairs cardiac function

Myofibroblast-mediated fibrosis in the myocardium is the hallmark of pathophysiological cardiac remodeling [13, 14, 18]. In addition to collagens, fibronectin and other structural ECM proteins, myofibroblasts also express a large number of matrix remodeling proteins and together determine the functionality of the ECM. Matrix metalloproteases (MMPs) are produced by myofibroblasts and are secreted as inactive zymogens that can be activated by cleavage of the propeptide, resulting in active proteases that can cleave collagens and other ECM proteins [2, 56]. These MMPs are negatively regulated by tissue inhibitors of metalloproteases (TIMPs), which are also secreted by myofibroblasts. TIMP1 is the major TIMP in the heart and is strongly induced in the failing heart [57]. Other matrix remodeling proteins that are strongly induced in the failing heart include, among others, lysyl oxidase-like 1 (LOXL1), latent-transforming growth factor beta-binding protein 2 (LTBP2), Gal-3, connective tissue growth factor (CTGF), periostin (POSTN), and Serpine2 [58]. Dysregulation of the proper balance between the different ECM and ECM-modulating proteins will affect the amount of connective tissue, its composition and quality, and will affect cardiac function. Recently, the plasma membrane glycoprotein syndecan-4 was identified to influence the degree of collagen cross-linking, which ultimately determined the degree of myocardial stiffness [59]. Syndecan-4 induces collagen, osteopontin (OPN) and lysyl oxidase (LOX) expression in cardiac fibroblasts and promotes LOX-dependent cross-linking of collagen fibers [59–61]. Thus, besides the amount of ECM, the level of collagen cross-linking also strongly determines the development of diastolic and systolic dysfunction [9, 16–18, 25, 56, 62].

Fibrosis also disturbs cardiac electrophysiology and induces rhythm disturbances. In mice, TGFβ1 overexpression resulted in atrial fibrosis, without ventricular involvement, and these mice developed inducible atrial fibrillation (AF) [63, 64]. Also in patients, AF is strongly associated with atrial fibrosis, [65] and it is now generally accepted that atrial fibrosis creates a substrate for AF [66, 67]. The mechanism by which myocardial scarring promotes rhythm disturbances is by creating reentry circuits [9, 16, 18, 62, 68, 69]. Furthermore, function of ion channels, ion pumps and gap junction proteins is also disturbed by cardiac remodeling [70]. Thereby, action potential conduction velocity is reduced which promotes susceptibility to reentry [70].

Finally, fibrosis limits nutrient supply toward the myocardium by limiting cardiac function and myocardial blood flow [9]. Perivascular fibrosis in coronary arteries reduces oxygen delivery toward myocardial tissue, reduces coronary reserves and promotes myocardial ischemia [16, 62]. Moreover, increased ECM deposition, which occurs often in association with hypertension and thus myocardial hypertrophy, results in diffusion problems in a situation in which demand for oxygen and nutrients is increased [14]. As discussed below, (myo)fibroblast also play an important role in cardiomyocyte hypertrophy development, and increased cardiomyocyte cell size will reduce oxygen diffusion in the cell interior. Decreased capillary density, as observed in heart failure tissue, will further contribute to cardiomyocyte dysfunction and finally cell death [71, 72].

Thus, fibrosis impairs cardiac function by at least three mechanisms, namely induction of myocardial stiffness, induction of AF and limiting oxygen and nutrient supply to the stressed myocardium. This will promote cardiomyocyte hypertrophy and cell death as schematically depicted in Fig. 2.

## Myofibroblasts and cardiomyocyte hypertrophy and cell death

Within the myocardium, extensive cross talk between cardiac fibroblast and cardiomyocytes via soluble factors and direct cell–cell interactions occurs [27]. Fibroblast–cardiomyocyte co-cultures and experiments with conditioned medium have shown that paracrine signaling from fibroblasts induces cardiomyocyte hypertrophy. A number of cytokines and growth factors, including TGFβ, interleukin-33 (IL-33), fibroblast growth factor 2 (FGF2), tumor necrosis factor alpha (TNFα), insulin growth factor (IGF1) and endothelin-1 (ET-1), are produced by (myo)fibroblast, which directly affect cardiomyocyte function in vitro and/or in vivo [27, 73–79]. Myofibroblasts contribute to the pathological hypertrophic response in cardiomyocytes via paracrine signaling, which ultimately can culminate in cardiomyocyte cell death. In mice with a fibroblast-specific knockout of Krüppel-like factor 5 (Klf5), a transcription factor required for the fibrotic response, both fibrosis and cardiomyocyte hypertrophy were strongly ameliorated in the pressure-overloaded heart. Moreover, pathological cardiomyocyte gene expression, as exemplified by Nppa expression, was strongly reduced in these mice [80].

In the last decade, a number of microRNAs (miRs) have been identified that are increased selectively in fibroblasts of the failing heart [15, 81]. The TGFβ-induced miR-21 inhibits sprouty homologue 1 (Spry1) expression, an endogenous inhibitor of the ERK-MAPK signaling pathway. This mechanism is important for fibroblast survival and for growth factor secretion and controls both the extend of interstitial fibrosis and cardiomyocyte hypertrophy. Silencing of miR-21 by a specific antagomir inhibited interstitial fibrosis and attenuated cardiomyocyte hypertrophy and cardiac dysfunction in a mouse pressure overload (transverse aortic constriction, TAC) model [82]. Surprisingly, a miR-21 knockout mouse model did not show diminished cardiac hypertrophy or fibrosis in response to pressure overload or angiotensin-2 (AngII) infusion [83]. This might be explained by compensatory mechanisms that are activated in the persistent absence of miR-21, and since HF is a chronic disease, this will likely limit therapeutic applications targeting miR-21 [83]. Another miR, miR-29, was found to be downregulated in fibroblasts after MI and controls expression of ECM genes [84]. Overexpression of miR-29b in the mouse heart prevented AngII-mediated cardiac fibrosis, cardiomyocyte hypertrophy and cardiac dysfunction [85]. As described above, myofibroblast depletion, by Gli1 cell ablation, also attenuated both fibrosis and cardiomyocyte hypertrophy after aortic banding, resulting in improved cardiac function [55].

Together these studies provide clear evidence that myofibroblasts are important drivers of pathological cardiomyocyte hypertrophy. Hypertrophic stimuli activate apoptosis signal-regulating kinase 1 (ASK1) in cardiomyocytes in vitro and in vivo, which induces both apoptotic and necrotic cell death [86, 87]. In ASK1 transgenic mice, induction of the pro-apoptotic protein Bax was reported [88]. In a hamster cardiomyopathy model, ASK1 inhibition by gene transfer of a dominant negative kinase into cardiomyocytes not only prevented apoptosis, but also chamber dilation and preserved left ventricular (LV) systolic and diastolic function. Moreover, cardiac interstitial fibrosis was significantly inhibited [89]. Cathepsin B (CTSB), a lysosomal cysteine protease, participates in apoptosis and autophagy and is expressed in murine and human hearts and induced by hypertrophic stimuli [90]. Not surprisingly, knockout of CTSB in mice attenuated pressure-overload-induced apoptosis, cardiac hypertrophy, fibrosis and cardiac dysfunction. Deletion of CTSB also prevented the activation of ASK1 and attenuated the release of cytochrome *c* from mitochondria, which has pro-apoptotic actions in the cytosol [90]. Interestingly, a direct role for CTSB in matrix remodeling in the eye has been described via the upregulation and/or proteolytic activation of ECM-remodeling enzymes [91]. Whether this is also true in the heart is not known, but it is tempting to speculate that certain proteins may directly link and coordinate fibrogenic and cell death processes. Nix, another factor with pro-apoptotic and necrotic actions in the heart, is induced by Gα_q_-mediated hypertrophic stimuli [92, 93]. Cardiac-specific Nix ablation prevented cardiomyocyte apoptosis and myocardial fibrosis and cardiac decompensation after TAC [94]. A recent co-culture study using adult rat fibroblasts, myofibroblasts and cardiomyocytes showed that both fibroblasts and myofibroblasts directly affect cardiomyocyte cell viability [95]. The TGFBR1 inhibitor SB341542 prevented this loss of cardiomyocyte viability, although this was most likely not a direct effect of TGFβ. It has been suggested that this might be mediated via TGFβ-dependent upregulation of ET1 in (myo)fibroblasts. The latter has been shown to stimulate cardiomyocyte apoptosis [96]. Thus, myofibroblasts are essential to induce pathological cardiomyocyte hypertrophy that may culminate in cardiomyocyte cell death via specific cell death signaling pathways, and, at least in vitro, myofibroblasts can directly promote cardiomyocyte cell death.

## The sustained fibrotic response

In normal wound healing, concomitant disappearance of myofibroblasts from the tissue marks the termination of the reparative response [97–100]. In many chronic diseases, including HF, a sustained fibrotic response is observed that further culminates in organ damage and finally organ failure. Apparently, at a certain stage of disease progression, a point of no return is reached and the fibrotic response cannot be downregulated anymore by the endogenous biological systems. The mechanisms behind this sustained response in the heart are multitude and include a positive feedback loop of fibroblast proliferation and FMT, inhibition of myofibroblast apoptosis, cardiomyocyte-mediated activation of fibroblasts and the presence of sustained low-grade systemic inflammation, as outlined below.

TGFβ plays an important role in fibroblast proliferation and FMT, and is produced in high amounts by myofibroblasts themselves resulting in a positive feedback loop. TGFβ therefore plays a central role in the sustained fibrotic response in the failing heart. TGFβ stimulates growth factor (EGF, IGF1)-mediated proliferation of fibroblast, and this probably also involves autocrine signaling via FGF2 and/or connective tissue growth factor (CTGF) [101, 102]. Inhibition of FGF2-induced fibroblast proliferation by TGFβ has also been described [103], indicating that this response is strongly dependent on the exact environmental factors. The increase in fibroblast numbers in the stressed heart indicates that in vivo this balance is shifted toward TGFβ-mediated fibroblast proliferation [49, 50].

TGFβ also prevents myofibroblast apoptosis via stimulation of PI3K/AKT pro-survival signaling pathway, and this is at least partly mediated via TGFβ-mediated secretion of ET1 [104]. The persistence of myofibroblasts can lead to nonresolving and progressive fibrosis, as exemplified by human idiopathic pulmonary fibrosis (IPF) [105, 106]. In pathological cardiac remodeling, persistent activation of the TGFβ pathway may therefore prevent myofibroblast apoptosis. This is consistent with the observation that myofibroblast can have very long life spans and continue secretion of pro-fibrotic factors and ECM proteins [107]. In vitro experiments using drugs targeting the TGFβ and MAPK pathways indicate that the myofibroblast phenotype can be reversed, but whether this also occurs in vivo and whether it can be stimulated in vivo is so far not known [108, 109].

In the heart, stressed cardiomyocytes themselves are important triggers of the fibrotic response of fibroblasts. Conditional, cardiomyocyte-specific knockout of Krüppel-like factor 6 (Klf6) in mice resulted in an attenuated fibrotic response after AngII infusion [110]. Surprisingly, a fibroblast-specific Klf6 knockout did not show this response. It was shown that Klf6 in cardiomyocytes controlled the expression of the ECM protein thrombospondin 4 (TSP4), which modulated activation of cardiac fibroblasts. This effect was specific for the AngII response and was not observed after aortic banding, indicating that under those conditions other factors are involved. Nevertheless, it indicates that as long as cardiomyocytes experience stress, these cells are able to produce factors that can induce and maintain a fibrotic response.

Inflammation is one of the main drivers of fibrosis and low-grade but persistent systemic inflammation is present in HF [111, 112]. Increased levels of cytokines and inflammatory biomarkers are present in patients, including TNFα, C-reactive protein (CRP), interleukin-6 (IL-6) and myeloid peroxidase (MPO), among others [113–117]. The precise mechanism of systemic inflammation in HF is unknown, but a growing body of evidence indicates that inflammation plays a role in the development and progression of HF and contributes to fibrosis. Cytokines, such as TNFα and interleukin-1β (IL-1β), are important activators of a variety of fibrotic diseases including cardiovascular diseases [118, 119]. TGFβ produced by myofibroblast may also play a role in cardiac inflammation. TGFβ has pleiotropic effects on the immune system and has both immunosuppressive and pro-inflammatory functions [120]. TGFβ can polarize macrophages and neutrophils toward a type II phenotype, which produces large quantities of inflammatory cytokines, including IL-6 and TGFβ. Inflammatory cytokines can activate cell death pathways and stimulate production of toxic reactive oxygen radicals (ROS) that can further exacerbate cardiac function. Moreover, systemic inflammation will not only affect myocardial function, but also other organs and therefore participate in the full manifestation of the complex HF syndrome [111].

## Targeting fibrosis

The above data show that (myo)fibroblasts and fibrosis adversely affect cardiac function and directly and indirectly contribute to cardiomyocyte death. This will further amplify this adverse cycle, leading to more fibrosis and cardiomyocyte death hereby contributing to HF development. Animal studies have shown that cardiac fibrosis appears not to be an essential stress response under many circumstances, but does impair cardiac function. Removal of the fibrotic trigger(s) would therefore constitute a potential way to halt the progression of cardiac remodeling and hence provide a cardiac sparing effect and promote cardiac function. This begs the question whether inhibitors of fibroblast activation or fibrosis can interfere with the fibrosis-cell death axis and thereby stop the vicious cycle which eventually leads to HF. Components of the cycle that are potential targets to interrupt the fibrosis-cell death axis are ECM proteins, paracrine and inflammatory signals, and fibroblasts/myofibroblasts.

By targeting ECM proteins and enzymes, such as MMPs, LoxL and Gal-3, the production and processing of collagen can be influenced. Several studies revealed that MMP inhibition results in less myocardial fibrosis and improves diastolic function [56, 121]. MMP inhibition results in less LV dilatation, while collagen accumulation does not occur [56]. Inhibition of LoxL reduces both the degree of myocardial collagen buildup and collagen cross-linking and is associated with less LV dilatation and preserved cardiac function in a mouse model of MI [122]. Inhibition of Gal-3, a fibrotic HF marker and a component of the ECM, resulted in less fibrosis and preserved cardiac function in animal models of HF [123]. These studies indicate that targeting ECM proteins holds promise to modulate the fibrotic response and HF progression.

Blocking paracrine and inflammatory signals can break the fibrosis-cell death axis as well. Several studies have investigated the effects of TGFβ pathway inhibition on fibrosis formation. In animal models of MI, early TGFβ receptor inhibition resulted in increased mortality, whereas late inhibition resulted in reduced fibrosis, improved contraction in the infarct zone and improved survival [44]. In pressure overload models, TGFβ receptor inhibition attenuated cardiac fibrosis and also preserved cardiac function [44]. However, TGFβ receptor inhibitors also induced valve lesions in animal studies and increased aortic rupture in a mouse pressure overload model [124, 125]. These TGFβ receptor inhibitors may therefore have limited clinical use for the treatment of chronic diseases. Treatment with pirfenidone, an anti-fibrotic drug for the treatment of IPF, also reduced fibrosis in a pressure overload mouse model of HF and preserved LV function [45]. The exact mode of action of pirfenidone is not known, but most likely interferes with TGFβ-induced fibrogenesis [45]. As revealed from animal studies, interleukin-1 (IL-1) is a mediator of fibrosis, by recruiting myofibroblasts and induction of MMP expression [126]. Clinical trials with IL-1 receptor antagonist treatments in MI patients did, however, not show beneficial effects on cardiac function [126]. The latter illustrates that, due to the complexity of cytokine actions and differences in the exact mechanisms between species, extrapolation to the human situation is not so simple.

The discovery of fibroblast-specific microRNAs that control the fibrotic response opens new avenues to directly target (myo)fibroblasts [15, 81]. Although constitutive downregulation of the fibroblast-specific miR-21 resulted in apparent fibrotic compensatory mechanisms [83], investigating other miRs could be more rewarding. A recent study showed that miR-125b is another critical regulator of cardiac fibrosis [127]. These authors showed that miR-125b is both necessary and sufficient for the induction of FMT via targeting of apelin, a critical repressor of fibrogenesis. In addition, miR-125b inhibited p53 expression, allowing induction of fibroblast proliferation. The clinical inhibition of miR-125b may therefore represent a novel therapeutic approach for the treatment of human cardiac fibrosis. Rather than downregulating miR expression using antagomirs or locked nucleic acid (LNA) oligonucleotides, overexpression of particular miRs should also be considered. Overexpression of the fibroblast miR-29b in the mouse heart prevented cardiac fibrosis [85]. Thus, clinical strategies to enhance miR-29b levels in patients could provide a promising strategy.

Another innovative strategy that holds great promise is the reprogramming of cardiac myofibroblasts into cardiomyocytes [128]. The regenerative capacity of the heart is thought to be minimal, but by reprogramming myofibroblasts into cardiomyocytes, the effects of the fibrosis-cell death axis could be reversed, thereby offering a curative approach for HF. Song et al. [128] showed that adult mice fibroblasts could be directly reprogrammed into cardiac-like myocytes in vitro and in vivo, using the transcription factors GATA4, HAND2, MEF2C and TBX5 (GHMT). Novel cardiomyocytes were indentified in infarcted areas of mice treated with intramyocardial injections of GHMT, and preservation of cardiac function was observed. This same group showed that human fibroblasts can similarly be reprogrammed in vitro toward a cardiomyocyte-like fate [129]. Generation of induced cardiac-like myocytes (iCLMs) could potentially be a promising new approach to regenerate lost cardiomyocytes [130]. Nevertheless, numerous technical and biological hurdles, including efficiency issues and the immature cardiomyocyte phenotype, need to be overcome.

## Discussion

**Fig. 3:**
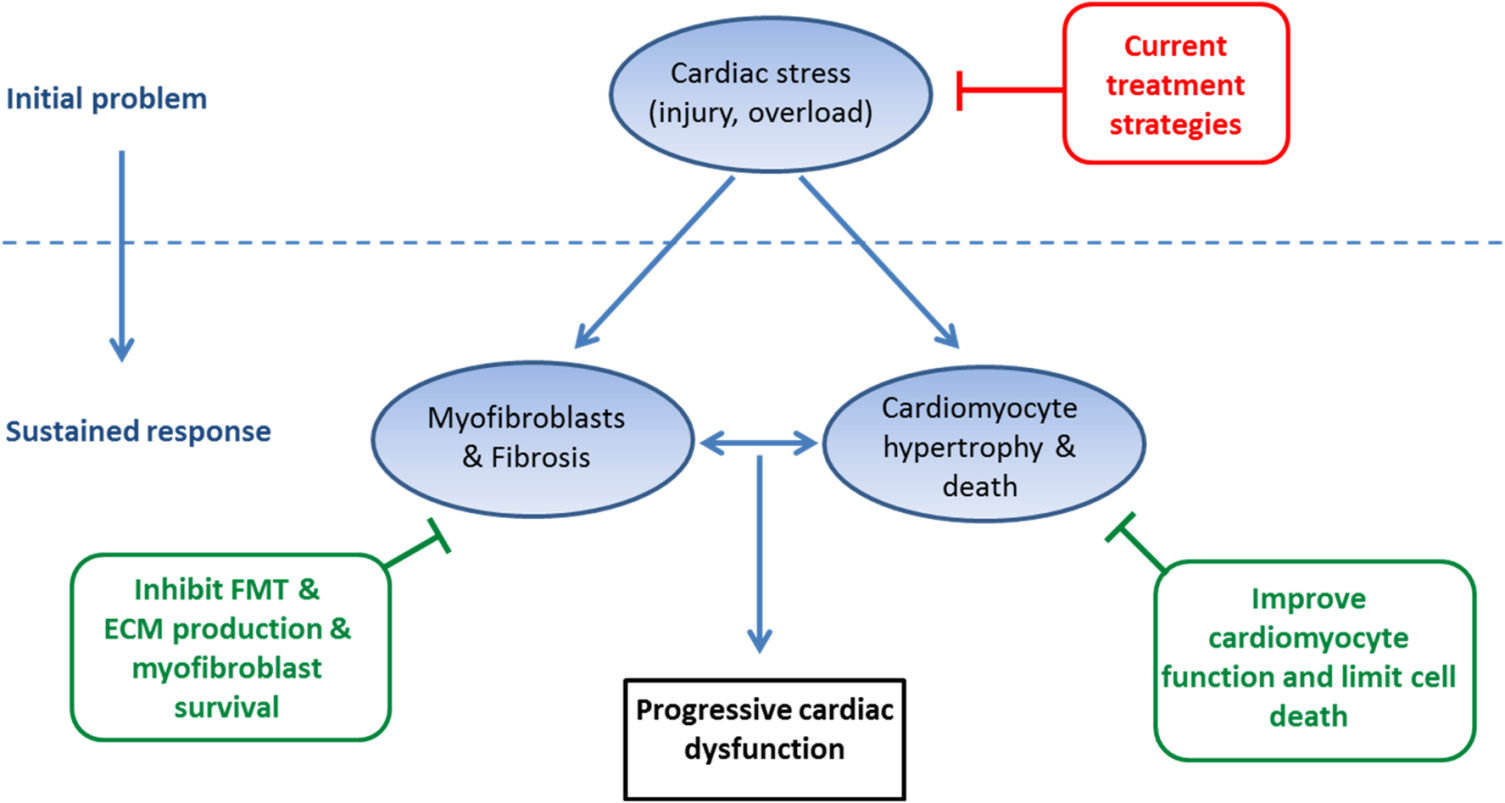
Suggested three pillars of heart failure treatment. Current standard heart failure (HF) therapy is focused at relief of the initial problem and aims at unloading the heart. Upon detection of HF, the sustained process of cardiac hypertrophy, fibrosis and cardiomyocyte cell death is already ongoing in most patients and cannot be stopped without additional treatment. We therefore suggest that to halt this sustained process, additional therapy will be required that blocks fibrotic processes and improves cardiomyocyte function. *FMT* fibroblasts to myofibroblasts transition, *ECM* extracellular matrix

HF is a major health problem in the Western world. Currently, the therapeutic approach toward HF mainly focuses on controlling symptoms and unloading the heart by reducing preload and afterload by treating HF patients with beta-blockers, angiotensin converting enzyme inhibitors (ACE-inhibitors), diuretics and aldosterone receptor antagonists [4, 5]. Although these drugs have shown beneficial effects in terms of relieving symptoms and reducing mortality [4, 5], prognosis for HF patients remains poor, with 5- and 10-year mortality rates of 50 and 90 %, respectively [131]. Current therapy reduces cardiac stress, but cardiac fibrosis is already present when most patients present themselves with symptoms at the clinic for the first time. Targeting the initial triggers, by unloading the heart, will slow down the vicious fibrosis-cell death cycle. However, it cannot eliminate the sustained fibrotic response present in the heart. Therefore, besides targeting the initial triggers, HF treatment should also focus on interrupting the fibrosis-cell death axis. As outlined in Fig. 3, we therefore believe that three pillars are important in HF therapy, namely unloading the heart, repressing fibrotic processes and improving cardiomyocyte function. Several ways to target myofibroblasts and fibrosis have been discussed above. We did not discuss the numerous studies that are aimed to improve cardiomyocyte function or limit cardiomyocyte death. Some interesting approaches that are under clinical investigation include the upregulation of the sarcoplasmatic reticulum calcium ATPase (SERCA2a) using gene therapy [132–134] and the use of myosin activators like omecamtiv mecarbil [135–137]. Whether apoptosis of cardiomyocytes can be inhibited at a clinical level, using caspase inhibitors or other cell death inhibitors needs to be awaited [138].

It is difficult to elucidate whether fibrosis or cardiac cell death should be marked as the driver of the fibrosis-cell death axis, but it seems likely that both are important players. However, what can be concluded is that myofibroblasts plays a central role in the fibrotic-cell death axis, by secreting ECM components and autocrine and paracrine signaling molecules that drive sustained fibrosis, cardiomyocyte hypertrophy and inflammation. More research will be needed to identify and test clinical approaches that can halt this vicious cycle of cardiac fibrosis and cell death in HF.
